# Antimicrobial resistance among people with intellectual disabilities in long-term care facilities: an exploratory, isolate-based surveillance study based on routine diagnostics between 2018 and 2023 in the Netherlands

**DOI:** 10.1093/jacamr/dlag124

**Published:** 2026-07-04

**Authors:** Soemeja Hidad, Wieke Altorf-Van Der Kuil, Freek De Haan, Geraline Leusink, Aura Timen, Annelot F Schoffelen, Sabine C De Greeff, Infectious Diseases Surveillance Information System-Antimicrobial Resistance (ISIS-AR) study group, J W T Cohen Stuart, D C Melles, K van Dijk, R P Schade, M Scholing, S D Kuil, A Alzubaidy, G J Blaauw, W Altorf—van der Kuil, S M Bierman, S C de Greeff, S R Groenendijk, R Hertroys, L Kruithof, I M Nauta, D W Notermans, J Polman, W J van den Reek, A F Schoffelen, C C H Wielders, B J de Wit, R E Zoetigheid, W van den Bijllaardt, E M Kraan, M B Haeseker, J M da Silva, E de Jong, B Maraha, M P A van Meer, A van Arkel, V Hira, A E Muller, M Wong, P Huizinga, E Bathoorn, M Lokate, J Sinnige, L E A Bank, F W Sebens, E Kolwijck, E A Reuland, S de Jager, M A Leversteijn-van Hall, J Gooskens, S P van Mens, E Schaftenaar, J C Rahamat-Langendoen, J M van Niekerk, P D J Sturm, B Wintermans, D S Y Ong, M van Rijn, S Dinant, M den Reijer, E I G B de Brauwer, A L E van Arkel, N van der Moeren, J J J M Stohr, A L M Vlek, F N J Frakking, K B Gast, H R A Streefkerk, S B Debast

**Affiliations:** Centre for Infectious Disease Control, National Institute for Public Health and the Environment, Bilthoven, The Netherlands; Department of Primary and Community Care, Radboud University Medical Center, Nijmegen, The Netherlands; Centre for Infectious Disease Control, National Institute for Public Health and the Environment, Bilthoven, The Netherlands; Centre for Infectious Disease Control, National Institute for Public Health and the Environment, Bilthoven, The Netherlands; Department of Primary and Community Care, Radboud University Medical Center, Nijmegen, The Netherlands; Department of Primary and Community Care, Radboud University Medical Center, Nijmegen, The Netherlands; Centre for Infectious Disease Control, National Institute for Public Health and the Environment, Bilthoven, The Netherlands; Centre for Infectious Disease Control, National Institute for Public Health and the Environment, Bilthoven, The Netherlands

## Abstract

**Background:**

Little is known about the presence of resistant micro-organisms in people with intellectual disabilities (ID) residing in long-term care facilities (LTCFs). We explored the resistant percentages of six highly resistant micro-organisms (HRMOs) among people with ID in ID-LTCFs: ESBL-producing *Enterobacterales* (ESBL-E), carbapenemase-producing *Enterobacterales* (CPE), *Acinetobacter* spp. (CPA) and *Pseudomonas aeruginosa* (CPPA), MRSA and VRE.

**Methods:**

We included data on the first isolate per species, per patient, per year ordered by ID physicians, as delivered by 11 clinical microbiology laboratories, participating in the Dutch National Infectious Diseases Surveillance Information System-Antimicrobial Resistance (ISIS-AR) between 2018 and 2023. Resistance was classified based on phenotypic and genotypic test results. The resistance percentage for each HRMO was calculated as the percentage of resistant isolates among the total number of isolates for each corresponding species.

**Results:**

The percentage of resistant ESBL-E isolates among *Enterobacterales* isolates was 3.2% (95% CI: 2.5%–4.0%), CPPA among *P. aeruginosa* isolates was 1.3% (0.4%–3.8%) and MRSA among *Staphylococcus aureus* isolates 0.4% (0.01%–2.35%). No CPE (n_tested_ = 2242), CPA (n_tested_ = 25) and VRE (n_tested_ = 6) were found.

**Conclusions:**

The resistance percentages for six HRMOs among positive diagnostic isolates ordered by ID physicians for people with ID in the Netherlands are low. The majority of HRMOs detected were isolated from older adults with ID. To estimate the burden of AMR and associated risk factors in the whole ID population, more extensive studies, including data from other medical specialties serving people with ID, such as general practitioners, are needed.

## Background

Antimicrobial resistance (AMR) has become a significant health threat with an estimated burden of over four million global deaths associated with bacterial AMR and over one million deaths directly attributed to bacterial AMR.^1,2^ Highly resistant micro-organisms (HRMOs) are of special concern as these micro-organisms cause infections that are difficult to treat and require additional infection control measures to prevent spread.^3^ In the Netherlands, the overall prevalence of HRMOs is low compared to most other countries.^4,5^ However, there are groups in the Netherlands, such as people with intellectual disabilities (ID) residing in long-term care facilities (ID-LTCFs), for whom the occurrence of AMR is unclear. Insight into the presence of AMR among people with ID in ID-LTCFs is important, as multimorbidity and health inequalities occur in this group.^6,7^

An ID is characterized by notable congenital impairments in both intellectual functioning and adaptive behavior.^8^ In the Netherlands, the prevalence of ID is estimated at approximately 1.5% of the total population.^9^ Although this comprises only a small proportion of the Dutch population, this group needs a relatively high amount of support and care, which is generally provided in specialized ambulatory and/or long-term care facilities (ID-LTCFs).^10^ Because ID can manifest in various ways, a range of care and housing situations exist, including living at home, in small community groups, or in ID-LTCFs.^11^ Full-time care services within ID-LTCFs are primarily used by people with moderate, severe or profound ID. In the Netherlands, medical care for the latter group is provided by a general practitioner (GP), whereas intellectual disability (ID) physicians, specialized in ID medicine, are mainly involved in disability related health issues such as epilepsy and syndrome-specific conditions. The ID physician is a medical specialism unique to the Netherlands that focuses exclusively on people with ID.^11,12^

Prior studies have shown that people with ID experience health-related problems more frequently than those without ID.^6,13,14^ For example, the COVID-19 pandemic disproportionately affected people with ID in terms of mortality, underscoring the population's vulnerability.^14^ In a setting with such vulnerable people, it is important to keep the prevalence of AMR as low as possible. There are currently no specific national guidelines in the Netherlands for routine HRMO screening in ID-LTCFs, apart from general HRMO screening recommendations (e.g. screening after submission in foreign hospital). Furthermore, to the best of our knowledge, no studies focusing on the prevalence of AMR within this population have been conducted until now. To address this gap, we conducted an exploratory retrospective study using routine diagnostic data of people with ID collected from clinical microbiological laboratories (CMLs) in the Netherlands. Using isolate-based surveillance data, we estimated the resistance percentages for six HRMOs included in the 2024 WHO Bacterial Priority Pathogens List: ESBL-producing *Enterobacterales* (ESBL-E), carbapenemase-producing *Enterobacterales* (CPE), carbapenemase-producing *Pseudomonas aeruginosa* (CPPA) and carbapenemase-producing *Acinetobacter* spp. (CPA), MRSA and VRE.^15^

## Methods

### Setting and data selection

We extracted data from the Dutch Infectious Disease Surveillance Information System—Antimicrobial Resistance (ISIS-AR), which collects antimicrobial susceptibility data from routine diagnostics performed by participating CMLs across the Netherlands.^16^ We included data on cultures that were documented as being ordered by ID physicians from 1 January 2018 to 31 December 2023, from CMLs that contributed during this period, regardless of whether they participated throughout the entire timeframe. Based on these inclusion criteria, data could be included from 11 CMLs serving multiple ID-LTCFs in the western, central and eastern regions of the Netherlands. Only isolates submitted by ID physicians were included, as they exclusively treat people with ID. Isolates submitted by other medical specialties such as general practitioners (GPs) were not included in this study, as we were unable to reliably identify isolates from people with ID in their records.

For the analysis, we selected the first positive diagnostic isolate (infection-related; i.e. cultures requested because of symptoms or clinical suspicion, not including screening cultures) per species, per patient, per year. We did not apply any selection based on specimen type.

In addition to the susceptibility test results, we received: year and month of birth, and sex of the patient from whom the isolate was obtained.

### Definition of HRMOs

We determined phenotypic susceptibility by reinterpreting the measured minimum inhibitory concentrations (MIC) values and diameters according to EUCAST breakpoints version 14.0 (2024).^17^ We estimated ESBL resistance percentages among *Enterobacterales* based on the results of phenotypical or genotypical ESBL confirmation tests provided by the CML. If these data were unavailable, we based the estimate on resistance to third-generation cephalosporins (cefotaxime/ceftriaxone, using a cut-off of 2 mg/L for both cefotaxime and ceftriaxone or 17 mm for cefotaxime and 22 mm for ceftriaxone and/or ceftazidime). Resistance percentage for CPE, CPA and CPPA was estimated using phenotypic carbapenemase-producing confirmation tests or genotypic molecular detection of carbapenemase genes. If we lacked data on such tests, we used resistance to meropenem and/or imipenem as a criterion: for CPE, we used resistance to meropenem (cut-off: 8 mg/L or 16 mm) and/or imipenem (cut-off: 4 mg/L or 19 mm); for CPA, resistance to meropenem (8 mg/L or 15 mm) and/or imipenem (4 mg/L or 21 mm); and for CPPA, resistance to meropenem (8 mg/L or 14 mm) and/or imipenem (4 mg/L or 20 mm). For MRSA, we based our estimation on phenotypic confirmation tests or genotypic molecular detection of *mecA* or *mecC* gene or *PBP2* expression in *Staphylococcus aureus*. If these data were unavailable, we used resistance to cefoxitin as determined by the CML. If cefoxitin susceptibility data were also missing, we used resistance to flucloxacillin or oxacillin as interpreted by the CML. Finally, we classified *Enterococcus faecium* isolates as VRE based on genotypic molecular detection of the *VanA* or *VanB* gene. When these data were unavailable, we classified isolates as VRE if they were resistant to both amoxicillin/ampicillin and vancomycin.

### Data analysis and data reporting

We conducted all data analyses in R version 4.4.0. We calculated descriptive statistics (n, %) for age group, sex, type of specimen and isolated species. Isolates with missing data for HRMO confirmation or susceptibility tests were excluded prior to resistance percentages calculation.

We report the resistance percentages of HRMOs as both the number (n) and percentage (%) of HRMO isolates among positive diagnostic cultures, together with a 95% Wilson CI. To calculate the percentages, we used the total number of isolates of the corresponding species or species group tested for the specific HRMO as the denominator. Within the ESBL-E species group, we additionally report the resistance percentages of two specific species: ESBL-producing *Escherichia coli* and ESBL-producing *Klebsiella pneumoniae*, as these are among the most commonly found in ESBL-related infections, such as urinary tract infections in community and healthcare settings.^5,18^

To assess whether the observed resistance percentages among isolates from positive diagnostic cultures were high, low or comparable to those in other Dutch health care settings, we compared our findings to data from Dutch general practices (representing the general population), and LTCFs (mainly nursing home residents) included in ISIS-AR.

### Ethical considerations

In accordance with national legislation, the Center for Clinical Expertise at the National Institute for Public Health and the Environment determined that approval by an ethical research committee or institutional review board was not required for this study (project number: EPI-703).

## Results

### Baseline characteristics of the data

We retrieved a total of 3372 isolates from 1654 patients, of which half were female. Patients aged 65 years or older represented ∼48% of all individuals, of whom a positive culture was included in our dataset. The majority of isolates were cultured from urine, followed by wound/pus/and skin cultures. The demographic characteristics of the included patients are shown in Table 1.

**Table 1.** Demographic characteristics of patients and origin of isolates for positive cultures ordered by ID physicians

Demographic characteristics of n = 1654 patients	n (%)
Gender	n = 1654
Female	841 (50.8)
Male	813 (49.2)
Age group in years	n = 1654
0–18 years	25 (1.5)
19–24 years	52 (3.1)
25–34 years	91 (5.5)
35–44 years	112 (6.7)
45–54 years	205 (12.4)
55–64 years	381 (23.0)
65–74 years	476 (28.8)
≥75 years	312 (18.9)
Origin of sample, number of isolates	n = 3372
Urine	2856 (84.7)
Wound/pus/skin	464 (13.8)
Lower respiratory tract	19 (0.6)
Genital	12 (0.4)
Higher upper respiratory tract	11 (0.3)
Others	10 (0.3)

From the 3372 isolates, *Enterobacterales* were the most prevalent (n = 2245, 66%), followed by *S. aureus* (n = 236; 7.0%), *P. aeruginosa* (n = 231; 6.9%) and *Acinetobacter* spp. (n = 33; 1.0%), see Table 2.

**Table 2.** Distribution of isolated micro-organisms by sampled material

	Total number of isolates, N (%)	Isolates from urine samples, N (%)	Isolates from wound, pus, skin samples, N (%)
*Enterobacterales*	2245 (66.6)	2077 (72.7)	149 (32.1)
*Staphylococcus aureus*	236 (7.0)	70 (2.5)	157 (33.8)
*Pseudomonas aeruginosa*	231 (6.9)	166 (5.8)	61 (13.1)
*Acinetobacter* spp.	33 (1.0)	26 (0.9)	7 (1.5)
*Enterococcus faecium*	6 (0.2)	6 (0.2)	0 (0.0)
Other micro-organisms^a^	621 (18.4)	511 (17.9)	90 (19.4)
Total	3372 (100)	2856 (100)	464 (100)

^a^Other micro-organisms include bacteria, yeasts and mixed flora. In order of frequency: These species were *Enterococcus faecalis* (174 isolates), *Aerococcus urinae* (121 isolates), *Streptococcus agalactiae* (74 isolates), *Enterococcus species* (34 isolates), *Streptococcus dysgalactiae n.n*.g. (33 isolates), *Aerococcus sanguinicola* (23 isolates), *Aerococcus viridans* (12 isolates). All other species were detected in 10 or fewer isolates, including a variety of Gram-positive and Gram-negative bacteria, yeasts and other micro-organisms.

Within *Enterobacterales*, *E. coli* accounted for half of the isolates (n = 1126; 50.2%), followed by *Proteus mirabilis* (n = 420; 18.7%) and *K. pneumoniae* (n = 280; 12.5%). Among urine samples, *Enterobacterales* were the most prevalent, accounting for two-thirds (n = 2077; 72.7%). Within this group, about half were *E. coli* (n = 1095; 53%). For wound/pus/skin samples, the most commonly found micro-organism was *S. aureus* (n = 157, 33.8%).

### Resistance percentages of HRMO in positive cultures requested by ID physicians for people with ID

The calculated resistance percentages of HRMO among positive diagnostic isolates can be found in Table 3.

**Table 3.** Estimated prevalence of HRMO (and 95% confidence interval) among positive diagnostic isolates from people with intellectual disabilities in ID-LTCFs in the Netherlands

HRMO/sample type	Number tested	Number positive	Prevalence (%)	95% CI
ESBL all sample materials				
ESBL-E (all *Enterobacterales*)	2031	64	3.2	2.5–4.0
ESBL-producing *E. coli*	1059	49	4.6	3.5–6.0
ESBL-producing *K. pneumoniae*	254	8	3.1	1.6–6.3
ESBL, urine				
ESBL-E	1876	58	3.1	2.4–4.0
ESBL-producing *E. coli*	980	45	4.6	3.5–6.0
ESBL-producing *K. pneumoniae*	244	7	2.9	1.4–5.8
Other HRMO, all sample materials				
CPPA (*P. aeruginosa*)	230	3	1.3	0.3–3.7
MRSA	229	1	0.4	0.01–2.35
CPE	2242	0	0.0	n/a
CPA	25	0	0.0	n/a
VRE	6	0	0.0	n/a

*Prevalence (%)* = number positive/number tested for corresponding species × 100%; 95% CI calculated using the Wilson method. For CPE, CPA and VRE, no positive isolates were detected. n/a = not applicable. For CPA, n = 8 were missing and for ESBL *Enterobacterales*, n = 214 were missing. For all other HRMO tests, missing results were <1%.

Out of 2031 *Enterobacterales* isolates with available ESBL test results, 64 (3.2%, 95% CI: 2.5%–4.0%) were classified as ESBL. The majority of ESBL-E isolates were *E. coli* (n = 49) and *K. pneumoniae* (n = 8). The other seven ESBL-E comprised *Klebsiella oxytoca* (n = 3), *Citrobacter amalonaticus* (n = 1), *Citrobacter freundii* (n = 1), *Enterobacter cloacae* complex (n = 1) and *Morganella morganii* (n = 1). Among *E. coli* isolates, the percentage of ESBL was 4.6% (49/1,059, 95% CI: 3.5%–6.0%). For *K. pneumoniae* isolates, the percentage of ESBL was 3.1% (8/254, 95% CI: 1.6%–6.3%). For urine samples specifically, we found an ESBL-E percentage of 3.1% among tested *Enterobacterales* (n = 58; 95% CI: 2.4%–4.0%).

The percentage of CPPA among isolates of *P. aeruginosa* was 1.3% (3/230, 95% CI: 0.3%–3.7%). The CPPA isolates were cultured from urine (n = 1), upper respiratory tract (n = 1) and lower respiratory tract (n = 1). For MRSA, we identified 1 positive isolate from urine (1/229, 95% CI: 0.01%–2.35%). We did not identify any *Enterobacterales* isolates meeting the CPE definition nor were any isolates detected that met the criteria for CPA or VRE (n_tested_ = 2,242 for CPE, n_tested_ = 25 for CPA and n_tested_ = 6 for VRE).

Missing data on relevant confirmation or susceptibility tests were relatively high for CPA (n = 8; ∼24%) and ESBL *Enterobacterales* (n = 214; ∼10%), with the majority of isolates with missing data originating from a single laboratory. Therefore, a sensitivity analysis was conducted excluding this laboratory, resulting in similar resistance percentages. For all other HRMOs, the percentage of missing data was low (<1%).

## Discussion

In this retrospective isolate-based study, we assessed the resistance percentages of ESBL-E, CPE, CPA, CPPA, MRSA and VRE among positive diagnostic isolates from a selection of people with ID in the Netherlands. Overall, resistance percentages for six HRMOs among isolates from positive diagnostic cultures requested by ID physicians were low, ranging from 0 to 4.6%. No CPA, CPE and VRE were found. These null findings do not imply absence of these HRMOs among people with ID in ID-LTCFs, but rather that they were not identified in our dataset.

The findings for ESBL *E. coli* (4.6%) are similar to those observed among diagnostic *E. coli* isolates from the Dutch GP patient population (4%), and in Dutch nursing home residents (∼5% resistance against third-generation cephalosporins for *E. coli*).^5^ ESBL *K. pneumoniae* in our study (3.1%) seems to be somewhat lower than the resistance percentages based on positive diagnostic isolates from the GP patient population (5%).^5^ For MRSA, our findings (0.4% in urine, and no isolates from wound/pus/skin cultures) were lower than in the GP patient population (4% in wound/pus/skin cultures) and Dutch nursing home residents (2% in wound/pus/skin cultures).^5^ These findings provide a first indication of similar or lower HRMO resistance percentages in the ID-LTCF population compared to the GP and nursing home patient population. The lower HRMO resistance percentages in ID-LTCFs are noteworthy, given that people with moderate to severe ID are assumed to be at higher risk for acquiring AMR bacteria, given their multimorbidity and frequent hospital admission.^6^ On the other hand, we assume that the risk of acquiring an HRMO through travel, which is a main risk factor in the Netherlands, is less likely among people with ID in ID-LTCFs compared to people without ID.^19–22^ Still, definitive conclusions on the presence of AMR in ID-LTCFs cannot be drawn from this study, since the number of isolates included was relatively small.

Previous carriage studies have reported ESBL prevalences ranging from 4–19% in Dutch nursing homes for older adults.^23–26^ Unlike carriage studies, our data are limited to infection-related samples (diagnostic) and only individuals with positive bacterial culture results are included (isolate-based surveillance) and we lack information on the total number of cultures performed (sample-based surveillance). Therefore, our estimates are likely an overestimation of the true resistance percentage, because cultures in first-line settings are generally obtained from patients with symptoms of infection who do not respond to initial antimicrobial treatment. Moreover, our findings are not directly comparable to findings from carriage studies, which also include asymptomatic carriers.

The data included in this study originated from CMLs located in western, central and eastern regions serving multiple ID-LTCFs. We assume similar variation between ID-LTCFs as seen in nursing home carriage studies, where ESBL and MRSA prevalence varied between facilities (range: 0%–15% for ESBL and 0%–9% for MRSA).^25^ However, due to the lack of detailed information on which ID-LTCFs are associated with the reported HRMO resistance percentages, we are unable to study whether such variation exists between ID-LTCFs.

The age and sex distribution in this study showed that 50.8% of patients were female, which is comparable to 44% in a Dutch nationwide ID prevalence study. The patients included in our study were predominantly older adults with ID. Remarkably, ∼48% of all positive bacterial cultures in our study came from patients aged 65 years or older. This is a larger percentage than expected, as only 8.5% of residents with ID in ID-LTCFs in the Netherlands are aged 65 years or older.^9,27,28^ If we compare our findings to the age distribution of positive bacterial culture results from the GP patient population, our study shows that the absolute number of isolates peaks in the 65–75 years age group, while in the GP patient population, the highest number is found in patients aged 75 years or older (Unpublished GP data in ISIS-AR; see Table S1 and Figure S1, available as Supplementary data at *JAC-AMR* Online). This difference in age distribution may be explained by earlier functional decline and greater frailty in people with ID, which is consistent with previous findings in this population.^27,29^ At the same time, both ID and GP patient populations show high proportions of older individuals with positive culture results, and for ID patients specifically, most ESBL isolates were retrieved from people with ID aged 65 years or older. This is in line with our expectation, as older age is known to be associated with an increased risk of colonization or infection with AMR. This in turn may overestimate the true prevalence of ESBL among all people with ID in ID-LTCFs, as older adults with ID are overrepresented in our dataset, and were shown to more frequently have ESBL-producing isolates.

The data from people with ID show that urine samples constituted the largest proportion of all submitted materials (∼85%), with *Enterobacterales* being the most frequently isolated species group. For wound/pus/skin cultures, *S. aureus* was the most isolated species (followed by *Enterobacterales*). This is in line with data from the GP and nursing home patient population.^5^ Interestingly, among people with ID in ID-LTCFs, *P. mirabilis* was found in 46% (n = 70) of all wound/pus/skin cultures from which *Enterobacterales* were isolated. This percentage is higher than that observed in similar cultures from GP patients, where *P. mirabilis* accounted for approximately 15% of *Enterobacterales* isolates.^5^ Reduced mobility and incontinence are important risk factors for intertrigo, which creates favorable conditions for skin infections like those caused by *P. mirabilis.*^30^ These risk factors are commonly found in specific subgroups of people with ID, especially those with an intellectual as well as a motoric disability.^31,32^ Although we cannot draw definitive conclusions since patient-level data on the presence of such risk factors were not collected, this may contribute to the increased occurrence of *P. mirabilis* skin infections in our study population.

### Strengths and limitations

One strength of our study is the exclusive inclusion of data from ID physicians. This provided an opportunity to examine microbiological data of people with ID. By using data from the Dutch national AMR surveillance database (ISIS-AR), where all information is handled uniformly, our results can be compared to resistance percentages in other healthcare settings in the Netherlands that submit their data to ISIS-AR.

Still, several limitations should be considered. First, we lack additional information on the ID physicians or associated ID-LTCFs. This limits the possibility to assess differences between specific ID-LTFs. Second, our results are based on data from 11 out of 48 CMLs, as only these laboratories recorded culture requests from ID physicians in a way that allowed us to reliably identify them. Other laboratories may also process culture requests from ID physicians, but due to differences in data registration and transfer to ISIS-AR, we could not recognize requests from ID physicians in their data. Third, it is unclear how resistance percentages might differ if data from other physicians (e.g. GP) or care settings were included. People with ID in ID-LTCFs for whom the GP is the primary care provider are not captured in our analysis. ID physicians are primarily involved in disability-related issues, but there is no indication that resistance patterns in bacterial infections diagnosed by ID physicians differ from those diagnosed by other physicians, such as the GP. Finally, our sample represents a fraction of ID-LTCFs, and there are known differences between Dutch ID-LTCFs in terms of AMR policies, antimicrobial stewardship (AMS) and infection prevention and control (IPC), which may affect the risk for acquiring AMR bacteria.^33^ Despite these limitations, the analysis provides a useful indication of resistance percentages for six HRMOs among positive cultures ordered by ID physicians in ID-LTCFs, but these findings cannot be extrapolated to the entire population of people with ID in the Netherlands.

### Conclusion

In this retrospective isolate-based surveillance study, we estimated the resistance percentages of six HRMOs among people with ID from ID-LTCFs in the Netherlands using data from the Dutch National Antimicrobial Resistance Surveillance System (ISIS-AR). By analysing AMR routine surveillance data specifically for this group, we provide new insight into the resistance percentages of HRMOs in a population for whom such information has rarely been reported. The estimated resistance percentages for ESBL-E, CPE, CPA, CPPA, MRSA and VRE in this study were low and do not suggest a higher risk for AMR among people with ID seen by ID physicians in the Netherlands.

To improve generalizability, future research should include larger samples and data from other medical specialties, such as GPs, as well as clinical and demographic information. This would help provide a more robust estimate of the HRMO resistance percentages and their associated risk factors in this population.

## Supplementary Material

dlag124_Supplementary_Data

## Supplementary data

Figure S1 and Table S1 are available as Supplementary data at *JAC-AMR* Online.
