# Supplementary material for: Antimicrobial resistance among people with intellectual disabilities in long-term care facilities: an exploratory, isolate-based surveillance study based on routine diagnostics between 2018 and 2023 in the Netherlands
Source: JAC Antimicrob Resist. 2026 Jul 4;8(4):dlag124. doi: 10.1093/jacamr/dlag124 (PMC13332802; doi:10.1093/jacamr/dlag124)
Supplement: dlag124_Supplementary_Data [file dlag124_supplementary_data.docx]

Supplementary file

**Table S1.** Age distribution from isolates in positive culture samples collected by general practitioners and intellectual disability physicians

| Age group | General practitioner patient population (GP)* | Prevalence (%) | Intellectual disability physician (IDP)* | Prevalence (%) |
| --- | --- | --- | --- | --- |
| 0–18 years | 111,481 | 11 | 25 | 2 |
| 19–24 years | 42,348 | 4 | 52 | 3 |
| 25–34 years | 79,706 | 8 | 91 | 6 |
| 35–44 years | 66,491 | 7 | 112 | 7 |
| 45–54 years | 89,350 | 9 | 205 | 12 |
| 55–64 years | 136,757 | 14 | 381 | 23 |
| 65–74 years | 190,935 | 19 | 476 | 29 |
| ≥75 years | 283,964 | 28 | 312 | 19 |

Based on ISIS-AR data from 2018–2023.
For each patient, only the first isolate over the entire period was selected, regardless of organism.
* IDP includes data from people with ID exclusively.

The data from the general practioner (GP) patient population covers all cultures from all CMLs that submitted data, so it is nationally representative. However, the prevalence of ID in the Netherlands is estimated at approximately 1.5% of the population, and it is important to note that a smaller percentage of individuals actually reside in ID-LTCFs from these 1.5%. Therefore, we expect that only a very small proportion of GP patient population data consists of people with ID who are not flagged (i.e., because the request was made by physicians other than ID physicians). As a result, we consider it unlikely that the potential inclusion of this small subgroup of people with ID in the GP patient data has a meaningful impact on the

comparisons or the overall interpretation.

Figure S1. Plotted age distribution from isolates collected from general practitioners (GP) and intellectual disability physicians (ID)


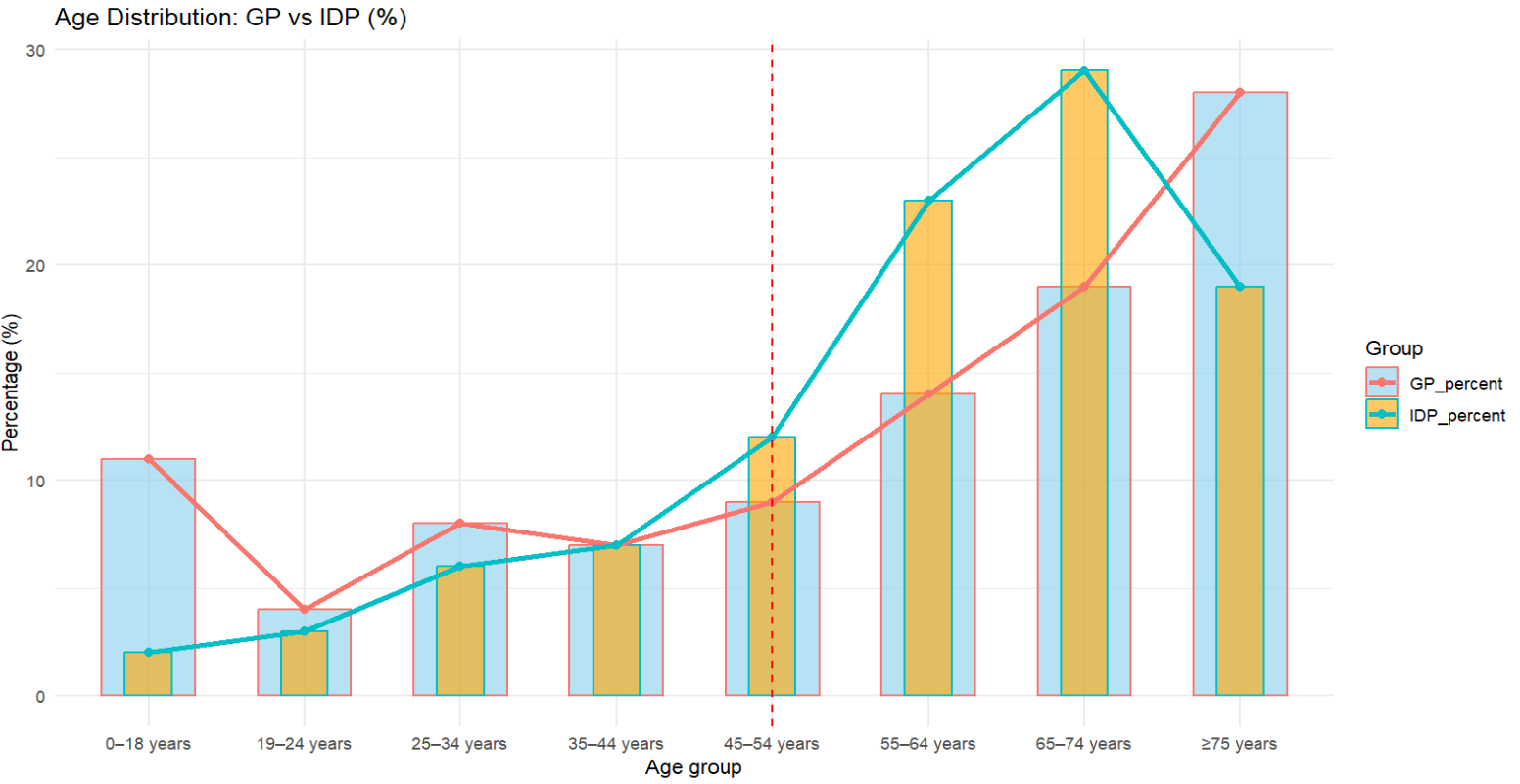


GP, general practitioner with data from the general population (people predominantly without ID); IDP, intellectual disability physician with data from people with ID. AST, antimicrobial susceptibility testing.

The figure demonstrates that, starting from the 45–54 year age group (red vertical dash line), there is a noticeable increase in the relative number of positive bacterial culture isolates with available AST results among people with intellectual disabilities (ID) compared to those who visit the general practioner (predominantly people without ID). This percentage peaks in the 65–74 year age group and subsequently declines in people aged ≥75 years. This pattern likely reflects the earlier onset of vulnerability and lower life expectancy among people with intellectual disabilities, resulting in fewer positive diagnostic isolates in the oldest age group.
